# SyNC, a Computationally Extensive and Realistic Neural Net to Identify Relative Impacts of Synaptopathy Mechanisms on Glutamatergic Neurons and Their Networks in Autism and Complex Neurological Disorders

**DOI:** 10.3389/fncel.2021.674030

**Published:** 2021-07-20

**Authors:** Rounak Chatterjee, Janet L. Paluh, Souradeep Chowdhury, Soham Mondal, Arnab Raha, Amitava Mukherjee

**Affiliations:** ^1^Department of Electronics and Telecommunication Engineering, Jadavpur University, Kolkata, India; ^2^SUNY Polytechnic Institute, College of Nanoscale Science and Engineering, Nanobioscience, Albany, NY, United States; ^3^Flash Controller Team, Memory Solutions, Samsung Semiconductor India Research, Samsung Electronics Co., Ltd., Bangalore, India; ^4^Advanced Architecture Research, Intel Intelligent Systems Group, Intel Edge AI, Intel Corporation, Santa Clara, CA, United States; ^5^Independent Researcher, Kolkata, India

**Keywords:** synaptic plasticity, software, hardware, synaptopathy, ASIC, computational neuroscience, accelerator

## Abstract

Synaptic function and experience-dependent plasticity across multiple synapses are dependent on the types of neurons interacting as well as the intricate mechanisms that operate at the molecular level of the synapse. To understand the complexity of information processing at synaptic networks will rely in part on effective computational models. Such models should also evaluate disruptions to synaptic function by multiple mechanisms. By co-development of algorithms alongside hardware, real time analysis metrics can be co-prioritized along with biological complexity. The hippocampus is implicated in autism spectrum disorders (ASD) and within this region glutamatergic neurons constitute 90% of the neurons integral to the functioning of neuronal networks. Here we generate a computational model referred to as ASD interrogator (ASDint) and corresponding hardware to enable in silicon analysis of multiple ASD mechanisms affecting glutamatergic neuron synapses. The hardware architecture Synaptic Neuronal Circuit, SyNC, is a novel GPU accelerator or neural net, that extends discovery by acting as a biologically relevant realistic neuron synapse in real time. Co-developed ASDint and SyNC expand spiking neural network models of plasticity to comparative analysis of retrograde messengers. The SyNC model is realized in an ASIC architecture, which enables the ability to compute increasingly complex scenarios without sacrificing area efficiency of the model. Here we apply the ASDint model to analyse neuronal circuitry dysfunctions associated with autism spectral disorder (ASD) synaptopathies and their effects on the synaptic learning parameter and demonstrate SyNC on an ideal ASDint scenario. Our work highlights the value of secondary pathways in regard to evaluating complex ASD synaptopathy mechanisms. By comparing the degree of variation in the synaptic learning parameter to the response obtained from simulations of the ideal scenario we determine the potency and time of the effect of a particular evaluated mechanism. Hence simulations of such scenarios in even a small neuronal network now allows us to identify relative impacts of changed parameters and their effect on synaptic function. Based on this, we can estimate the minimum fraction of a neuron exhibiting a particular dysfunction scenario required to lead to complete failure of a neural network to coordinate pre-synaptic and post-synaptic outputs.

## 1. Introduction

The exceptional computational power of the brain in memory and learning is accomplished by conversion of an electrical signal into the interneuronal transmission of chemical neurotransmitter information at synapses (Hebb, 1949; Burns and Augustine, 1995; Südhof and Malenka, 2008). As the complexity of synapses continues to evolve, so must the evolution of computational and hardware tools to evaluate such models. The events are non-spontaneous and dependent on input intensity and frequency and the measured electrophysiological response is an action potential that brings into play the complex physiology of neuron dendritic, cell body, axonal hillock, and axonal compartments. The largest contribution of information passing in neural circuits occurs at synapses (Di Maio, 2008) and is regulated by a diversity of synaptic plasticity mechanisms (Citri and Malenka, 2008; Choquet and Triller, 2013) that must operate over timescales (Abbott and Regehr, 2004). In 1949, Hebb described activity-dependent synaptic modulation (Hebb, 1949) that forms the basis of current models of neuronal plasticity. Long-term changes impact learning and memory and short-term changes support synaptic computations (Tetzlaff et al., 2012). This can be described in terms of spike timing dependent plasticity (STDP) that relates changes in synaptic strength, or synaptic weights, to the timing of pre- and post- synaptic spikes, a key mechanism in memory formation. When linked to synaptic stability it can be used to describe flexible or stable memories (Park et al., 2017) and complex topologies of neural networks (Borges et al., 2017; Lameu et al., 2021). Less investigated in regard to learning and memory is the relative impact of altered biological mechanisms on synaptic strength, owing to lack of generalized computational models as well as scalable hardware architectures that can process such complexity.

As the main information transfer between neurons, neurotransmitter release is a highly regulated yet probabilistic process (Benfenati, 2007; Regehr, 2012). Indeed in short term plasticity to modify neuronal circuits, synapses are viewed as active filters of information, not just conveyers, reducing noise and enhancing relevant information (Klyachko and Stevens, 2006). An important mechanism to dynamically fine tune the probability of neurotransmitter release is through local feedback regulation (Branco and Staras, 2009; Minneci et al., 2012). Intermediate initial release dynamics behave as band pass filters. These events and others allow adaptive regulation to changes in network activity and enables neurons to respond to prolonged alterations. How relative changes to this homeostatic mechanism arising from synaptopathies affect synaptic plasticity has not been previously examined. Here we consider the glutamatergic synapse, that is a low pass filter type. Glutamatergic neurons encompass most of the synapses in the central nervous system (CNS) and are relevant to cognitive decline (Volk et al., 2015). We generate a neuron model that is adapted to study the implications to synaptic strength and efficiency when stochastic variations occur in underlying mechanisms and that can also be implemented into hardware.

Spiking Neural Networks (SNNs) are used to model synapses to reflect action potential spikes and a large number of SNN software and hardware models have been proposed for mimicking neurological behavior. The software based SNN neural analysis methods that have been developed include NEURON (Hines and Carnevale, 1997), Brain (Goodman and Brette, 2009), NeMo (Fidjeland et al., 2009), PyNN (Davison et al., 2008), and more. Although providing biological accuracy, these software based simulations encounter extraordinarily high computational costs for subsequent hardware development while performing numerical simulations. Hence modern computers fail to obtain real-time performance when scaled to simulate large neural networks. As a case in point, a 1 s simulation of a network composed of 8 million neurons that includes 4 billion integrate-and-fire synapses when analyzed on the Gene rack supercomputer using 2,048 processors, takes ~80 min. By co-development of software and hardware implementation jointly, considerations of compactness, power-efficiency, and ease of implementation of circuits can be optimized. In this regard, circuits involving feedback mechanisms are desired and has led to analog circuit designs of SNNs. The relative efficiency of an analog vs. digital implementation varies dependent on the required signal-to-noise ratio. In neural dynamics challenging temporal features are present, such as high and nonuniform pulse latency and activity dependent synaptic plasticity that results in long-lasting long-term potentiation (LTP) processing. This creates a need for capacitors of value >0.1 F, that imposes physical constraints. This challenge is significant and nullifies much of the advantage offered by analog circuits in terms of a smaller number of transistors. Analog circuits also suffer from transistor mismatch, process variation and model size limitations owing to a process termed gate fan-out that relates the number of gate inputs to a single original logic gate. Another challenge is in reliable analog memory, that has not yet been robustly achieved in regard to storage of significant processing values. This includes synaptic weight whose subtle variations have significant importance to the accuracy of the entire system. Hence, owing to essential needs in user capabilities, including resource management and real-time speed, we employ a digital solution for large-scale simulation of neural networks. Our approach emulates neuron spiking with minimum possible error and also importantly without sacrificing speed of processivity of the digital system implemented as hardware.

A number of synaptic models have been developed for digital implementation including the Time Machine approach, SpinNaker (Furber et al., 2014), Neurogrid (Benjamin et al., 2014), and BrainScales (Pfeil et al., 2013), among others. BrainScales is a wafer-scale neuromorphic system, in which each wafer contains 48 reticles with eight High-Count Analog Neural Network (HiCANN) dice. Each such HiCANN die has the capability to emulate 512 adaptive exponential neuron models. SpiNNaker is a one-million-core supercomputer, developed exclusively for massively-parallel real-time simulation of large-scale neural networks, making it one of the largest digital neuromorphic platforms to date. Neurogrid is a real-time system consisting of over one million quadratic integrate-and-fire neurons, in which neuron and synapse dynamics are emulated using analog circuits and communications are performed by digital means, for simulation of over a billion synapses. However, since these models have been developed with the intention of utilization in Spiking Neural Network (SNN) architectures, they only approximate the effect of the numerous parameters in a neuron.

In current large scale neuromorphic platforms that rely on SNN architectures, the exclusive abstraction of intracellular dynamics of a neuron that ignores other biologically modulated parameters is severely restricted in applications to understand and modulate plasticity. Our jointly developed software and hardware solutions are designed instead to be able to retain computational speed while also simulating the effect of a broader range of individual biologically relevant parameters in the neuronal synapse and its networks relevant to normal function and synaptopathies. Glutamate is known to be the main excitatory neurotransmitter in the CNS and accounts for 90% of the total neurotransmitter usage in the CNS. Glutamate provides us with the most fundamental form of a neuron and is ideal for extension to other models. In developing the hardware architecture Synaptic Neuronal Circuit, SyNC, we focus on the design of the excitatory glutamatergic neuron communication network. Synaptic plasticity and multiple brain functions rely on glutamate that is the most abundant excitatory neurotransmitter in the brain. Glutamate and glutamatergic neurotransmission dysfunction is central to ASD (reviewed in Rojas, 2014) and to broader impacts on neurodegeneration (Lewerenz and Maher, 2015) as well as psychiatric disorders (Li et al., 2018). To capture the speed of the network we use a retrograde messenger mediated plasticity (RMMP) model as opposed to a glial cell-mediated model (Postnov et al., 2007). In the RMMP bipartite neuron system there are no intermediary dynamics considered in the synaptic cleft and hence the synaptic current from the pre-synaptic region is the same as the synaptic current entering the post-synaptic region. We consider the dynamics of retrograde messengers that travel across the synapse via diffusion to be the feedback initiator in our model. These considerations allow us to expand biological detail in a digital framework to address multiple pre- and post-synaptic interacting molecular mechanisms found in synaptopathies and to cross-evaluate dysfunction by a rapid multi-classification comparison. Expanded detail includes receptor inhibitors and activators, including allosteric regulation, as well as receptor type ratios, synaptic cell adhesion molecules, and calcium signaling and organelle stores.

**Figure 1:**
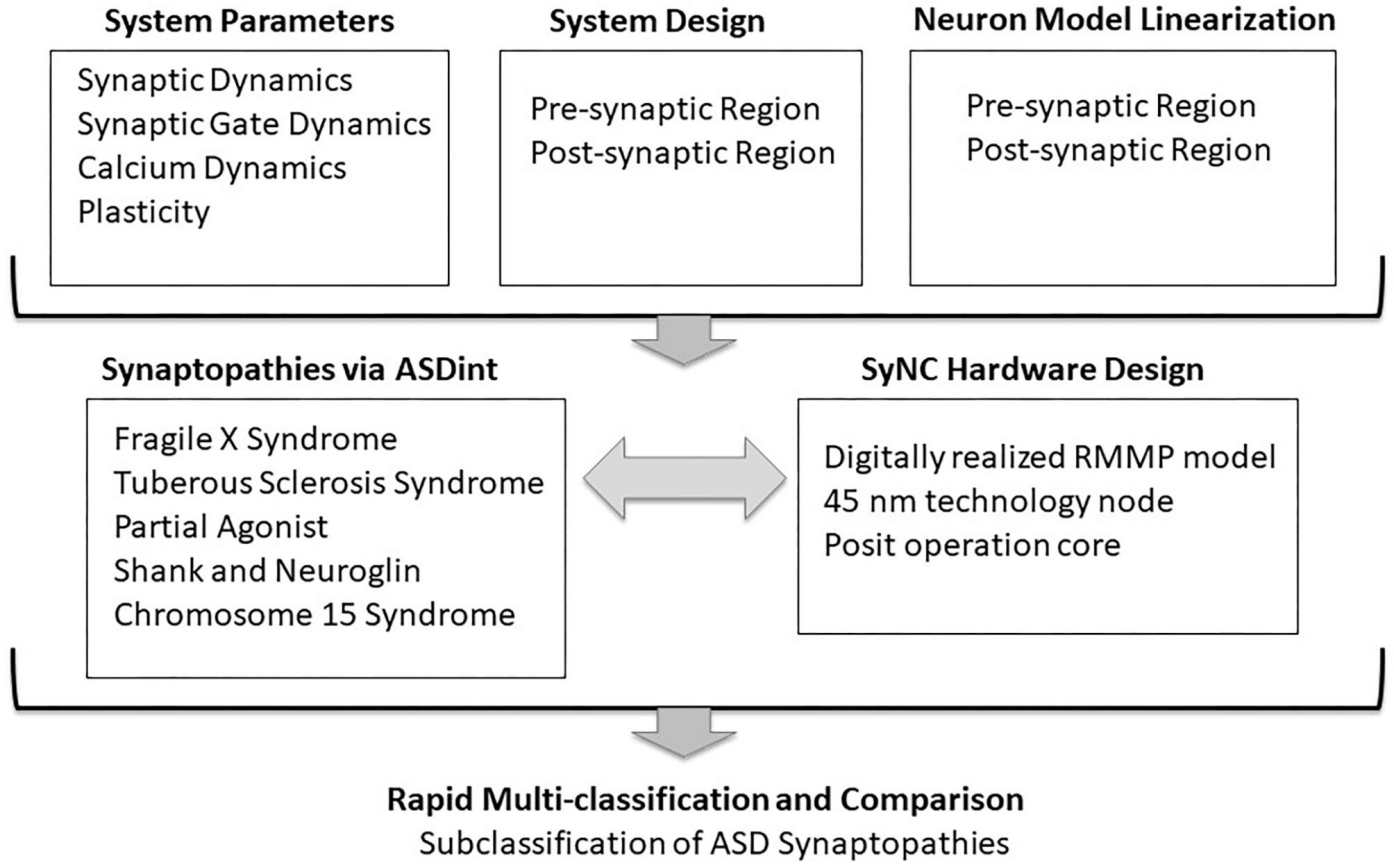
Co-development of SyNC with neural algorithms to relay ASD complexity. The goal of this study was to advance algorithms of neuronal synapse modeling in the context of a complex neural disorder, such as ASD, while obtaining high (1 GHz) optimal frequency in hardware. The diagram outlines general considerations in codevelopment. mGluR, metabatropic glutamate Receptor; NMDAR, N-methyl-D-aspartate Receptor.

In our model, we consider Hebbian plasticity parameter Δ which records the activity at pre-synaptic and post-synaptic boutons and evaluates the gain in synaptic potential in the form of synaptic weight. While many different mechanisms have been suggested in the literature to explain LTP, we have approached it from a rather simplistic model such that it can be adapted for utility in other models as well. To our knowledge this is the first work to co-develop and implement a diverse and highly adaptable design of a neuron in detail in software and hardware and we demonstrate its effectiveness in complex and scaled neuronal networks applied to Autism Spectrum Disorders (ASD) (Figure 1). We mapped and synthesized our SyNC hardware designs in 45 nm technology. Our simulation results show that differential forms of these equations reproduce the expected characteristics of the pre-synaptic region of a neuron, while conveniently transformed into discretized form. Application of ASDint over the SyNC core demonstrates an ability to effectively reproduce phenotypes for each synaptopathy. Synthesis results show that using a posit operation core gives us PPA performance that is slightly better than the IEEE 754 double precision while having higher accuracy than the IEEE single precision operation core. This outcome affirms the use of posit as a valid replacement of floats. In relation to biological discovery, the SyNC platform for the first time, allows us to examine complex individual and combined impacts of synaptopathies on synaptic plasticity in real time.

## 2. Background

### 2.1. System Description

#### 2.1.1. The Dynamic Synapse as a Lipid-Derived Retrograde Signaling Model

**Figure 2:**
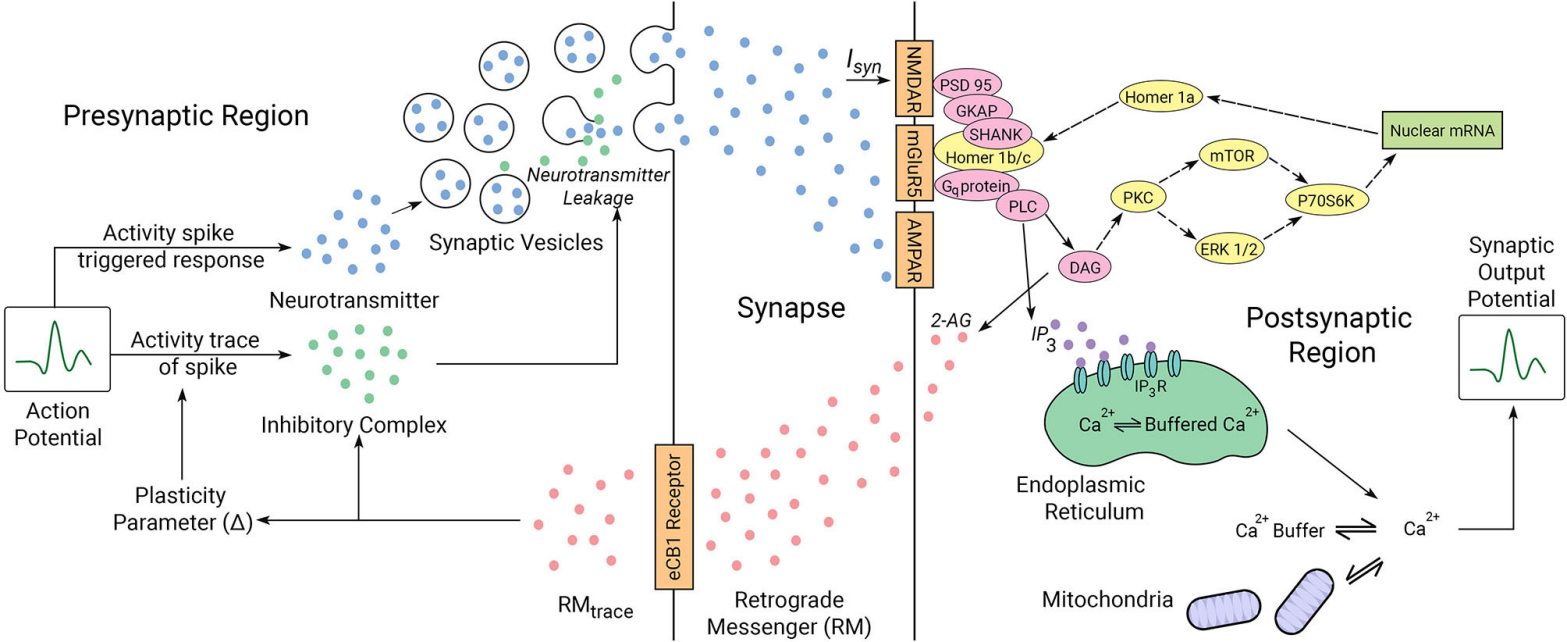
Illustrated mechanics of the neuron system model. The pre-synaptic and post-synaptic region dynamics are described for the model. The synaptic cleft acts as a lossless channel, and is not involved in any dynamic processes involving neuronal communication. Proteins expressed in yellow are involved in the interactions between the locally transcribed mRNA and the synaptic gate receptors as indicated by dotted arrows. Proteins represented in pink are bridge proteins involved in inter-receptor dynamics. The parameters involved in synaptic activity are linked by solid arrows. The roles of the proteins in synaptic activity diagrammed here are described in the text in regard to ASD mechanisms we have considered.

How to best incorporate the interplay between synapse dynamics that is generated by multiple underlying interacting mechanisms (Choquet and Triller, 2013) and which defines plasticity remains a challenge. In Figure 2, we describe the key elements in our proposed synaptic dynamics model. Two primary components in our software for synapse dynamics are retrograde signaling and post-synaptic receptor type. Retrograde signaling (Brenman and Bredt, 1997; Regehr et al., 2009) systems integrate the post-synaptic response with regulation of pre-synaptic output generating rapid changes in synaptic strength. Although diverse chemical messengers exist, retrograde models share many basic steps. Firstly, the production and release of retrograde messengers from post-synaptic cells are regulated by post-synaptic calcium, and triggered by post-synaptic metabotropic receptors and second messenger. Secondly, the entirety of the concentration of retrograde messenger produced at the post-synaptic region is assumed to be transmitted to the pre-synaptic receptors with no losses in transmission or within the synaptic cavity. While such an assumption might feel out of place, we consider that such losses if they happen initially would eventually saturate to a point in which no loss occurs. The retrograde messenger acts on the pre-synaptic target to modulate the plasticity parameter and activity spike strength in the pre-synaptic region.

The duration and latency of a retrograde signal is controlled by parameters of uptake and degradation of the molecules at a cellular level. As a result, the nonlinear relationship between the release of a retrograde signal and the magnitude and duration of the retrograde signal available to activate pre-synaptic receptors must be considered in the ultimate effect on synaptic strength. A final consideration is the extent to which the release of the retrograde messenger can be sustained. Many cells contain few vesicles and dense core secretory granules in their dendrites, which suggest that it would be possible to deplete the release of conventional neurotransmitters, peptides, and growth factors. The low density of vesicles suggests that the dendritic release may be much more prone to depletion. The ability to recover from depletion would then depend crucially on endocytosis and vesicle and granule refilling. Alternately, some lipid-derived messengers are produced on demand (Piomelli, 2003; Chevaleyre et al., 2006), and as a result the release of these messengers may be sustained. To describe the regulated release of neurotransmitter at the synapse, we consider the lipophilic (lipid-derived) retrograde signaling model (Chevaleyre et al., 2006). The output of our model for observing effects of plasticity is calcium dynamics in the pre-synaptic region and therefore we can determine the impact of all the processes in the model that impact that parameter. Plasticity is achieved in the system by increasing the latency of calcium signals, which in turn can lower the peak calcium levels. Activation of Gq-coupled receptors such as mGluR1 in the post-synaptic region can promote release of inhibitory factors in the pre-synaptic region by increasing the production of retrograde messenger 2-AG that in turn reduces calcium levels.

#### 2.1.2. Synaptic Gate Dynamics and Glutamatergic Receptors

Amongst the six main neurotransmitters in the human body, the main excitatory neurotransmitter in the brain is glutamate, which activates several postsynaptic receptors. Two primary types of receptors encountered in a glutamatergic neuron are ionotropic Glutamate Receptors (iGluR) and metabotropic Glutamate Receptors (mGluR). The mGluRs bind glutamate within a large extracellular domain and transmit signals through the receptor protein to intracellular secondary messenger signals. The receptors primarily involved in this process are the group I mGluRs: mGluR1 and mGluR5. Ionotropic glutamate receptors (iGluRs) are faster responding and the two major types of iGluRs: α-amino-3-hydroxy-5-methyl-4-isoxazolepropionic acid (AMPA) and N-methyl D-aspartate (NMDA) have central roles in hippocampal synaptic plasticity. Both are ligand-gated ion channels and have unique properties that subserve different phases of synaptic plasticity. Glutamate released from the pre-synaptic neuron opens AMPA receptors to depolarize the post-synaptic cell. Each AMPA receptor has four sites to which an agonist (such as glutamate) can bind, one for each subunit. The channel opens when two sites become occupied, and current increases with subsequent binding. The AMPA receptor's permeability to calcium, and other cations such as sodium and potassium, is governed by the presence of the GluA2 subunit in the AMPARs. The presence of GluA2 renders the channel impermeable to calcium and is proposed to guard against excitotoxicity.

#### 2.1.3. Calcium-Mediated Synaptic Events Including Organelle Stores

Calcium mediated synaptic events have been proposed to sustain temporary holding of information as in working memory (Mongillo et al., 2008) and is considered in our model. To enable the flow of synaptic current through the post-synaptic receptors, mGluR1-type receptors coupled to the G protein (Gq) are activated. Together calcium and Gq activate phospholipase C beta (PLC), which cleaves the lipid phosphatidylinositol bisphosphate (PIP2) into diacylglycerol (DAG) and inositol trisphosphate (IP3). DAG is converted into the endocannabinoid 2-arachidonoylglycerol (2-AG). The rate-limiting and Ca2+-sensitive step in 2-AG production is the formation DAG. IP3 and DAG are free to diffuse through the cell cytoplasm and their impacts can be described computationally. In our model we consider the role of mitochondria and the endoplasmic reticulum (ER) in calcium dynamics. Mitochondria are important for numerous roles related to synaptic transmission and neurodegeneration (Lee et al., 2018) including calcium regulation in neurons (Gunter and Gunter, 1994; Gunter et al., 2004) along with the ER (Karagas and Venkatachalam, 2019). Calcium mobilization from mitochondria is controlled by neurotransmitter release (Rizzuto et al., 2003) as well as more complex proposed buffering roles (Matthews and Dietrich, 2015).

When IP3 binds to an IP3 receptor (IPR) on the ER membrane it causes the release of *Ca*^2+^ from the ER. Five pathways have been considered in the modulation of *Ca*^2+^ influx, which we describe in a pair of dynamic equations. Thus, we efficiently incorporate abstractions of several terms in our model (Figure 2) to describe these dynamics. *Ca*^2+^ taken up into (Juni), or released from (Jmito) mitochondria, and that bound to (Jon), or released from (Joff), *Ca*^2+^ buffers are considered to create a constant *Ca*^2+^ flow that does not vary with instigation of cell membrane receptors. 2-AG then regulates pre-synaptic pathways to affect synaptic transmission by decreasing the probability of neurotransmitter release from the pre-synaptic terminal. In the model, the common cell structures act as an impedance and the calcium buffers in the cytoplasm provide capacitance and are not a part of the signaling circuit, hence acting as a channel to drain higher frequencies away.

### 2.2. Application of Anti-symmetric Hebbian Plasticity

Synaptic strength is influenced by pre- and post-synaptic activity in activity-dependent synaptic plasticity processes such as long-term potentiation LTP and LTD (Lee et al., 2013). Hebbian plasticity is used to define these features in synaptic plasticity (Hebb, 1949). The N-methyl-D-aspartate receptors (NMDARs) are calcium permeable and when activated, allow an influx of calcium needed for the induction of LTP. However, NMDARs require both pre-synaptic transmitter release and post-synaptic depolarization for activation. Enhancement in the amplitude of action potential takes place when both the pre-synaptic and post-synaptic regions are active, resulting in potentiation of synaptic output potential. However, when either region is selectively active, the amplitude of action potential is attenuated, resulting in depression of synaptic output potential. These two processes together constitute the synaptic plasticity mechanism and hence act as substrates for fundamental brain function. LTP is a process involving such persistent enhancement of synaptic gain resulting in a long-lasting increase in synaptic transmission gain between the neurons. It is an important process in the context of synaptic plasticity. LTP recording is widely considered to be the cellular model for storage of information in the brain. LTD is an opposite process that modulates and controls the effect of LTP in the brain. Full opening of the NMDAR channel and the consequent influx of calcium requires both the binding of glutamate to the receptor and post-synaptic depolarization. The induction of potentiation is dependent on activation of NMDARs and a rise in post-synaptic calcium. The NMDAR dependence provides a ready explanation for the associativity and asymmetry of Hebbian learning rule. The binding of glutamate follows the release of a transmitter by the pre-synaptic spike, and the post-synaptic depolarization is provided by the post-synaptic spike. Thus, neither the release of glutamate alone nor the post-synaptic spike alone will result in the opening of the receptor. Both must occur at the same time. In the frog optic tectum (Zhang et al., 1998) and in cultured hippocampal cells (Bi and Poo, 1998), no potentiation was observed when the pre-synaptic spike preceded the post-synaptic spike by more than 20 ms and no depression was observed when the pre-synaptic spike followed the post-synaptic spike by more than 20ms. LTD is induced at a lower concentration of calcium than required for induction of LTP, however the parameters of the timing window for depression are not fully predicted by the expected calcium concentration alone. In layer V/VI of the neocortex of the developing frog optic tectum (Zhang et al., 1998) and cultured hippocampal cells (Bi and Poo, 1998), the depression, like the potentiation, depended on the activation of NMDARs, but the depression found in layer II/III pyramidal cells of the somatosensory cortex did not. It is observed for layer II/III pyramidal cells that the interval for depression is considerably larger than the interval for potentiation. All of these learning rules are asymmetric, in that positive actions have different effects than negative delays. Tsodyks and Markram (1997) proposed an anti-symmetric form of Hebbian plasticity where the time interval of potentiation and depression are comparably similar. This is the form of plasticity we have used in our system.

### 2.3. Modeling Neuronal Damage Scenarios Associated With Autism Spectrum Disorder

**Figure 3:**
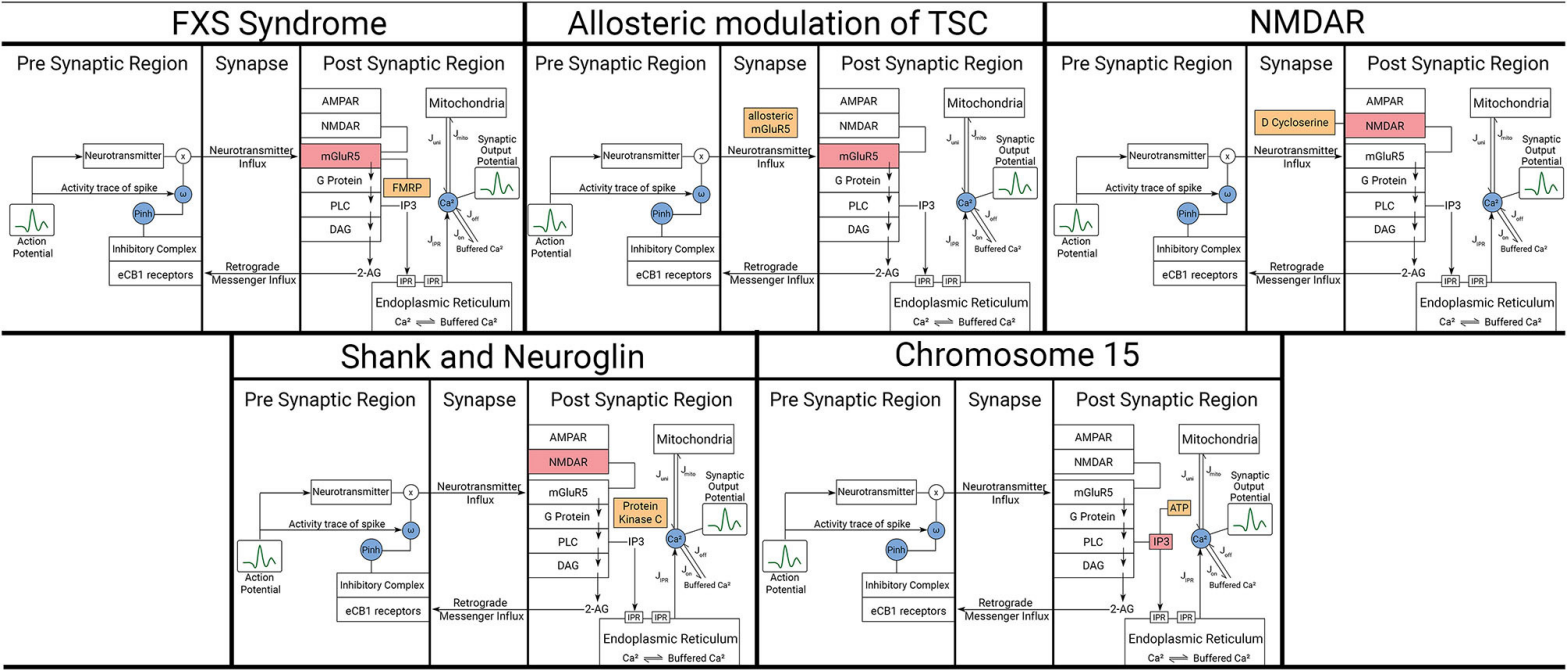
Representation of affected components and modulators in the model for each considered ASD scenario. The five scenarios here are described in detail in the text. Orange boxes represent the modulating variables and red boxes represent the components being affected by those variables. Blue circles represent mathematical variables describing certain phenomenon in those pathways.

ASDs form a group of diverse neurodevelopmental conditions defined by two core symptoms: social deficits that include communication and interaction impairments; and stereotypical, repetitive, and restricted behaviors. One of the main features of ASD is the high level of heterogeneity resulting in complexity both when considering symptoms and causative factors. It is in fact possible that every single disorder within the group has its unique mechanisms and consequences, with environmental and genetic factors playing roles in the etiology of ASD. In that regard, computational models will be imperative to help compare and categorize ASD effects on synaptic function. We consider multiple ASD mechanisms. It is in fact possible that every single disorder within the group has its unique mechanisms and consequences, with environmental and genetic factors playing roles in the etiology of ASD. We consider multiple ASD mechanisms in mathematical descriptions that incorporate various regions of the synapse (Figure 3).

#### 2.3.1. Fragile X Syndrome

For our model, we consider the role of Fragile X protein, FMRP, in mRNA translation regulation and binding, which is decisive in determining the quality of synaptic communication between the pre-synaptic and post-synaptic regions (Davis and Broadie, 2017). It has been recently demonstrated that alterations in signaling, expression, and function of group I mGluRs are related to neurodevelopmental disorders (Wang et al., 2018). Group I mGluRs comprising mGluR1 and mGluR5 have been proposed as key regulators of syndromic and non-syndromic forms of ASD, making them possible therapeutic targets. There are two distinct groups of mGluRs. Some groups of mGluR couple with Gq proteins and are involved in excitatory neural communication, like mGluR1 and mGluR5. The rest of the mGluR groups are involved in non-excitatory neural communication without assistance from Gq proteins. mGluR5 signaling was shown to be affected in opposing directions in both Fragile X Syndrome (FXS) and Tuberous Sclerosis Complex (TSC). We observe the response of these variations onto secondary mediator dynamics in our model, which is directly related to mGluR5 dynamics. Activation of group 1 mGluR mediates the release of *Ca*^2+^ in the postsynaptic region through the activation of kinases such as mTOR and ERK. These kinases are involved in the modulation of Homer 1a protein generation in the nucleus, which in turn modulates the mGluR5 receptor dynamics through its companion protein Homer 1 b/c. FMRP in this mechanism plays the role of a inhibitor responsible for attenuating the production of Homer 1a. However, in the absence of FMRP, there is an abnormal increase in mGluR5 receptor dynamics. The consequent excessive mGluR-LTD constitutes the aberrations observed in FXS.

FXS is the most common monogenic form of inherited intellectual disability. In a majority of the cases observed, the cause of hampered FMRP dynamics is the expansion of the CGG trinucleotide, repeating in the five untranslated region of the Fragile X FMR1 gene. When the repetition of CGG is as high as more than 1,000 times, this segment of the FMR1 gene undergoes methylation, effectively silencing gene activity and consequently, the generation of FMRP protein. FMRP suppresses mGluR5 receptor dynamics in the post-synaptic region and absence of FMRP leads to exaggerated synthesis of proteins required for mGluR dependent LTP, thereby enhancing its magnitude.

#### 2.3.2. Tuberous Sclerosis Syndrome

Another inherited intellectual disability associated with ASD is Tuberous Sclerosis Syndrome (TSC). The syndrome is caused by heterozygous mutations in genes of the TSC1/2 complex involved in mTOR mediated signaling that couples cell surface receptors to protein synthesis (Kelly et al., 2018). Several studies of mouse models of TSC in mouse models have exhibited reduction in decreased mGluR5-mediated LTD caused by impaired protein synthesis. Consistently, treatment with an allosteric mGluR5 agonist was able to restore mGluR5-mediated LTD (Auerbach et al., 2011). Both positive and negative allosteric modulation of mGluR5 have been proposed as a therapeutic intervention in neurodevelopmental disorders. Negative allosteric modulators (NAMs) of mGluR5 have been shown to alleviate long-term memory deficits, excessive repetitive behaviors, motor stereotypes and social interaction abnormalities in various models of autism (Silverman et al., 2012; Tian et al., 2015). The mGluR5 positive allosteric modulator (PAM) was reported to ameliorate deficits in learning and memory and chemically induced hippocampal LTD in Tsc2+/mice.

#### 2.3.3. NMDAR and the Partial Agonist D-Cycloserine

The NMDAR is a glutamate channel protein and ion receptor at the synapse. The implication of NMDARs in the etiology of ASD has been supported by both clinical and non-clinical studies. Clinical studies have identified genetic variants in the GRIN2A and GRIN2B genes encoding the GluN2A and GluN2B subunits of the NMDAR, respectively. It is highly plausible that differences in subunit composition affect functional properties of NMDARs and/or NMDAR-dependent plasticity. A role for NMDARs in ASD is supported by the fact that social withdrawal and repetitive behavior in individuals with ASD can be alleviated by the NMDAR co-agonist D-cycloserine. By contrast, NMDAR antagonists memantine and amantadine improve ASD-related symptoms. Thus, ASD could result, at least in part, from deviations in hormetic NMDAR responses. D-cycloserine is considered to be a partial agonist of NMDAR i.e., it acts like an agonist when it is the primary neurotransmitter involved but has antagonistic features when it is abundant in the system. This seems to be due to its different receptor subtype selectivity and intrinsic action, which depends on various NR2 subunits (NR2A, NR2B, NR2C), which happen to be the location of glutamate binding. One of the most prevalent hypotheses suggested is that the effects seen *in vivo* at low doses of D-cycloserine reflect its agonistic action at the NR1/NR2C receptors, for which it has a high affinity, while at high doses the effects might be due to antagonistic inhibition of NR1/NR2A and NR1/NR2B receptors, for which D-cycloserine has a lower affinity. It is observed that this effects the glutamate binding and hence does not affect the plasticity from its own path. The resultant glutamate binding is inhibited at NR2A, NR2B once all sites of NR2C are occupied by either of the agonist. It must be noted that D-cycloserine in natural state is not an activated pathway, hence making it the noise source in the system.

#### 2.3.4. Shank and Neuroglin

Shank proteins are master scaffold proteins within the post-synaptic density of glutamatergic neurons important for synaptogenesis and function (Sala et al., 2015) but have also recently been described to have a pre-synaptic role in Drosophila (Wu et al., 2017). Mutations in human Shank genes are also found in ASD (Durand et al., 2007; Berkel et al., 2010; Sato et al., 2012) and expected to relate to their role in activity-dependent formation and remodeling of synaptic function. Analysis in a mouse model of Shank2, lacking exons 6 and 7, showed reduced hippocampal NMDAR function, whereas mice lacking only exon 7 show up-regulation of NMDARs in synaptosomes, increased NMDAR/AMPAR ratio, and enhanced NMDAR dependent LTP (Wegener et al., 2018). We can model this condition by varying the equilibrium constant value of k or kNMDAR. Hence, the synaptic weight limit is increased as the limit of excitation current is increased. Due to higher NMDAR/AMPAR value, we adopt a working assumption that synaptic vesicle release is unbounded. However, two Shank2 deletion mouse models resulted in very similar social deficits, which support the notion that deviation in NMDAR function in either direction can result in ASD like phenotypes. Interestingly, aberrant NMDAR function and behavioral deficits observed in those mice could be normalized with systemic D-cycloserine and administration of the positive modulator of mGluR5 3-Cyano-N-1, 3-diphenyl-1H-pyrazol-5-ylbenzamide (CDPPB) (Jiang and Ehlers, 2013). Shank1 similarly has been associated with ASD-like behavior in mice, including increased anxiety and deficits in contextual fear learning, but with improvements in spatial learning. This corresponds well with the cognitive changes observed in many individuals with ASD. Shank1 mice additionally show smaller dendritic spines in CA1 pyramidal hippocampal neurons and weaker synaptic transmission (Hung et al., 2008), supporting recent evidence that dendritic spine abnormalities are associated with ASD. We also consider Neuroglins that are synaptic cell adhesion molecules (Dean and Dresbach, 2006; Craig and Kang, 2007) in which several mutations have been found associated with ASD (Südhof, 2008). Knocking-in the ASD-associated R451C substitution into the endogenous Neuroglins NLGN3 locus caused a prominent decrease in Neuroglin levels that resulted in impaired social behaviors, enhanced spatial learning, and increased synaptic inhibition in the mouse somatosensory cortex. Its effects are complementary to Shank, as in the ratio of AMPAR/NMDAR becomes lower. We model the Shank and Neuroglin mechanisms on the effective change in synaptic current and the impacted changes can be observed in the secondary mediator dynamics.

#### 2.3.5. Chromosomal Defects

##### 2.3.5.1. Chromosome 15 Syndrome

A “chromosome 15 phenotype” characterized by ataxia, language delay, intellectual disability, repetitive movement disorders and facial dysmorphic features has been described in individuals with chromosome 15 duplications (Muhle et al., 2004). Within the 15q11–15q13 locus. We model the chromosome 15 inhibitory receptor action on neuronal dynamics onto the synaptic mediator dynamics and observe their effect on the calcium dynamics in the post-synaptic region of the neuron as a consequence of ITP3K enzyme and ATP on IP3 dynamics.

## 3. Methods for Implementation of Software and Hardware Models

### 3.1. Algorithm Design

#### 3.1.1. Pre-synaptic Region

We consider the neuron model proposed by Faghini-Moustafa (Faghihi and Moustafa, 2015) that describes the processes involved in synaptic plasticity in a synapse mediated by retrograde messengers (RMs). The model, described in Figure 2, demonstrates the synaptic current release from the pre-synaptic region when activity spike and retrograde messenger are provided to it. The amount of RM that diffuses into the pre-synaptic neuron consists of RMtrace. Activity trace of input spike considers the effect of plasticity and assigns the effect of latency to the input impulse train. RMtrace along with the activity trace of input spike decide the amount of inhibitory complex released. This inhibitory complex decides the effective release of synaptic vesicles from the pre-synaptic region. Thus, inhibitory complex concentration along with the concentration of neurotransmitter release and the activity trace of input spike decide the magnitude associated with the released synaptic current. This value is the synaptic weight and its rate of change gives the synaptic efficacy, a parameter that describes the strength of plasticity.

The equilibrium point of inhibitory complex is adjusted because the value considered in Faghihi and Moustafa (2015) has a very high threshold and is impractical, to a value where the effects of the impulses can be observed distinctly.

The state variables of the pre-synaptic region: effective strength of feedback of Retrograde Messenger (RMtrace), Inhibitory complex concentration (Inh), and activity trace of spike (C), and the concentration of neurotransmitter (D) are defined using the Tsodyks Markram Model as:

(1) $$\begin{array}{l} \frac{d}{dt}RMtrace=-\frac{RMtrace}{\tau_{r}}+RM \end{array}$$

(2) $$\begin{array}{l} \frac{d}{dt}Inh=-\frac{Inh}{\tau_{inh}}+RMtrace.C \end{array}$$

(3) $$\begin{array}{l} \frac{d}{dt}C=-\frac{1}{\tau_{c}}\left[C+\Delta \delta \left(t-t_{p}\right)\right] \end{array}$$

(4) $$\begin{array}{l} \frac{d}{dt}D=-\frac{1}{\tau_{d}}\left[D+\sum \delta \left(t-t_{d}\right)\right] \end{array}$$

where, τ_*r*_ is the time constant associated with the influx of RMs, τ_*inh*_ is the time constant associated with inhibition of release of neurotransmitters, *t*_*p*_ is the time when an impulse is received at the pre-synaptic neuron, RM is the concentration of retrograde messenger present in the post-synaptic region and τ_*c*_ and τ_*d*_ are the time constants associated with the biological latency in activity spike and neurotransmitter release, respectively. Equations (1)–(3) give the resulting effect of the action potential on the concentration of the inhibitory complex. Equation (4) gives the concentration of neurotransmitter released from the pre-synaptic neuron which depends on the rate at which impulses enter. Δ is the parameter for Hebbian plasticity and is calculated according to Table 1.

**Table 1:** Specific values of timescales, threshold values, and control parameters in neuron model.

**u[n]**	***sgn*(*RMtrace*−*RMrest*)**	**Δ**
1	1	1
1	0	−1
0	1	−1
0	0	0

The probability of inhibition and release of neurotransmitter, concentration of neurotransmitter and synaptic current are written as:

(5) $$\begin{array}{l} P_{inh}=e^{e^{-\frac{0.0000001}{Inh}}-1} \end{array}$$

(6) $$\begin{array}{l} P_{rel}=P_{init}\left(1-P_{inh}\right) \end{array}$$

Here, the probability of inhibition is a function dependent only on the concentration of the inhibitory complex. The probabilities considered here decide the synaptic weight of a neuron i.e., the amount by which the transmission of an impulse through the synapse is magnified. The rate of change of synaptic weight is called synaptic efficacy and is calculated as:

(7) $$\begin{array}{l} \frac{d}{dt}\omega =P_{rel}.C.D \end{array}$$

Hence, the resulting synaptic current transmitted can be written as:

(8) $$\begin{array}{l} Isyn\left(t\right)=\omega \sum \frac{t-t_{p}}{\tau }e^{\frac{t-t_{p}}{\tau }}\delta \left(t-t_{p}\right) \end{array}$$

#### 3.1.2. Post-synaptic Region

At the post-synaptic RM, the RM depends on the calcium concentration due to endoplasmic reticulum and secondary mediator. Calcium effects due to endoplasmic reticulum and secondary mediator can be written as:

(9) $$\begin{array}{l} \tau_{C_{c}}\frac{dC_{c}}{dt}=-C_{c}-C_{4}.f\left(C_{c},C_{e}\right)+[r+\alpha \left(W_{post}-W_{post_{rest}}\right)\\ +\beta S_{m}]+k.\frac{dm}{dt} \end{array}$$

(10) $$\begin{array}{l} \tau_{C_{c}}.e_{C_{c}}\frac{dC_{e}}{dt}=f\left(C_{c},C_{e}\right) \end{array}$$

(11) $$\begin{array}{l} f\left(C_{c},C_{e}\right)=C_{1}\frac{C_{c}^{2}}{1+C_{c}^{2}}-\frac{C_{e}^{2}}{1+C_{e}^{2}}.\frac{C_{c}^{4}}{C_{2}^{4}+C_{c}^{4}}-C_{3}C_{e} \end{array}$$

(12) $$\begin{array}{l} \frac{dm}{dt}=v_{1_{max}}\frac{C_{c}^{2}}{K_{d}^{2}+C_{c}^{2}}-v_{2_{max}}\frac{{\left[Na^{2+}\right]}^{2}}{K_{Na^{2+}}^{2}+{\left[Na^{2+}\right]}^{2}}.\frac{m}{1+m} \end{array}$$

(13) $$\begin{array}{l} \tau_{Sm}\frac{dSm}{dt}=\left(1+tanh\left(S_{Sm}\left(I_{syn}-I_{Sm}\right)\right)\right)\left(1-Sm\right)-\frac{Sm}{d_{Sm}} \end{array}$$

Here, in these equations, c1, c2, c3, and c4 are the fixed control parameters of the function f(cc,ce), cc describes the calcium concentration in the cytoplasm, ce represents the calcium concentration in the internal store (endoplasmic reticulum ER), Wpost is the recovery variable for the post-synaptic current in FHN model, Sm is the generation of IP3 in response to the influx of calcium current. The term [*r* + α(*Wpost* − *Wpostrest*) + β.*Sm*] represents the calcium influx from the external space. Also, interaction between the cytoplasmic calcium (cc) and endoplasmic calcium (ce) is described with a two-variable function f(cc,ce). There is threshold value for the Sm production that is triggered by the synaptic current. Threshold parameter ISm is hence selected to distinguish between activated and inactivated states of the variable.

The plasticity of the system is decided by the retrograde messenger. The concentration of retrograde messenger is only dependent on the post-synaptic calcium concentration. The concentration of post-synaptic retrograde messenger is written as:

(14) $$\begin{array}{l} RM=e^{-e^{-e\sqrt{3}log\left(C_{c}\right)+25.4146}} \end{array}$$

By FHN model, a simplified version of the Hodgkin-Huxley model, for post-synaptic site, we get:-

(15) $$\begin{array}{l} \frac{dW_{post}}{dt}=V_{post}+I_{post}-I_{syn}\\ \frac{dV_{post}}{dt}=V_{post}-\frac{V_{post}^{3}}{3}-W_{post} \end{array}$$

### 3.2. Linearization of Neuron Model

The equations are optimized to improve the computational efficiency of the model and reduce its implementation cost by implementing polynomial expansion of functions to reduce complex and lengthy functions. While this does mean that the hardware will be easier to design, the approximations are taken such that they resemble the curve closely in the domain the functions shall be operating in.

#### 3.2.1. Pre-synaptic Region

In the 1st order Tsodyks Markram differential Equations (14)–(4), since all the equations are linear, there is no requirement to make any adjustments to them.

For determining the probability of inhibition, we encounter the implementation of exponential function. On using CORDIC algorithm to implement the same, the area of the hardware is increased by a large amount. Also since the CORDIC algorithm operates on convergence, this would lead to an increase in time taken per process for computation. Hence, we solve by using an rectangular hyperbolic function that closely resembles the curve in the region of operation.

(16) $$\begin{array}{l} P_{inh}=\frac{-0.00007}{Inh-0.00001}+1.1 \end{array}$$

For synaptic current, we consider the summation segment as a reset enabled function. Thus, we obtain a pair of equations Y and Z, which generate the form of unweighted synaptic current.

(17) $$\begin{array}{l} Y=\frac{t-t_{p}}{\tau }e^{\frac{t-t_{p}}{\tau }}\\ Z=\tau e^{\frac{t-t_{p}}{\tau }} \end{array}$$

For a pre-fixed frequency of *t*_*p*0_ interval, the summation symbol in (8) is removed without any loss of accuracy by considering the summation of each power for a finite number of terms. However, for SyNC model to function for any form of *t*_*p*_ provided, we needed to make some approximations. Adoption of a piece-wise approach leads to a trade-off between area efficiency and error efficiency. Also the properties of the curve are lost at every new impulse completely, which leads to significant error in output, unless more functions are added to compensate the loss. But this almost doubles the area. Hence, the alternative is to go for differential form of these equations. As such the impulse driven activation can be enabled, without removing the value of Y completely before the activation. For this particular set of equations, activation conditions can be met simply by adding 1 to Z, when an impulse arrives in each cycle. Such a form is more robust in error handling for discretized calculations. Based on above technique, Isyn is calculated as follows:

(18) $$\begin{array}{l} \frac{dY}{dt}=\frac{Z-Y}{\tau }\\ \frac{dZ}{dt}=\frac{-Z}{\tau }+u\left[n\right] \end{array}$$

(19) $$\begin{array}{l} Isyn=\omega .Y \end{array}$$

#### 3.2.2. Post-synaptic Region

It is observed that the value of f(*c*_*c*_, *c*_*e*_) is in the order of 10–12, hence making the detection of this potential via instruments impossible. Moreover, the value of the function is much lower than the noise threshold, due to which the noise parameter will be the primary contributor to the value of this function. Hence, we consider f(c,E)=0. This in turn gives us E=0 (from Equation 12). Hence, the changed calcium dynamics equations are:

(20) $$\begin{array}{l} \tau_{C_{c}}\frac{dC_{c}}{dt}=-C_{c}+\left[r+\alpha \left(W_{post}-W_{post_{rest}}\right)+\beta S_{m}\right]+k.\frac{dm}{dt} \end{array}$$

(21) $$\begin{array}{l} \frac{dm}{dt}=v_{1_{max}}C_{c}^{2}K_{d}^{2}\left(1-\frac{C_{c}^{2}}{K_{d}^{2}}+\frac{C_{c}^{4}}{K_{d}^{4}}\right)-v_{2_{max}}.k1.\left(1-m+m^{2}\right)\\ \;k1=\frac{{\left[Na^{2+}\right]}^{2}}{K_{Na^{2+}}^{2}+{\left[Na^{2+}\right]}^{2}} \end{array}$$

For secondary mediator, polynomial expansion of tanh is implemented to reduce the complexity of the equation:

(22) $$\begin{array}{l} \tau_{Sm}\frac{dSm}{dt}=\left(1+k_{s}-\frac{k_{s}}{3}\right)\left(1-Sm\right)-\frac{Sm}{d_{Sm}}\\ \;k_{s}=S_{Sm}\left(I_{syn}-I_{Sm}\right) \end{array}$$

For concentration of retrograde messenger, we implement polynomial expansion of exponential function:

(23) $$\begin{array}{l} RM=1-\left(k2.c^{k1}\right)+\frac{{\left(k2.c^{k1}\right)}^{2}}{2}-\frac{{\left(k2.c^{k1}\right)}^{3}}{6} \end{array}$$

where k1 = 2.0447, k2 = 9.1799*x*10^−12^.

We modify the original FHN model such that the nonlinear terms are eliminated. Unlike the other equations, where we have allowed powers of a variable in expansion, we avoid this scenario here since we are able to reduce power taken in this particular variable description with no noticeable precision loss. This enables us to approximately solve both linearized FHN equations for the power cost of one. So, the nonlinear term from equation:

(24) $$\begin{array}{l} f\left(V_{post}\right)=V_{post}-\frac{V_{post}^{3}}{3} \end{array}$$

can be rewritten into the closest fitting linear curve (Hayati et al., 2016). The curve we chose here is:

(25) $$\begin{array}{l} f\left(V_{post}\right)=1-\frac{\left|V_{post}\right|}{2} \end{array}$$

## 4. Results

### 4.1. Hardware Design

**Figure 4:**
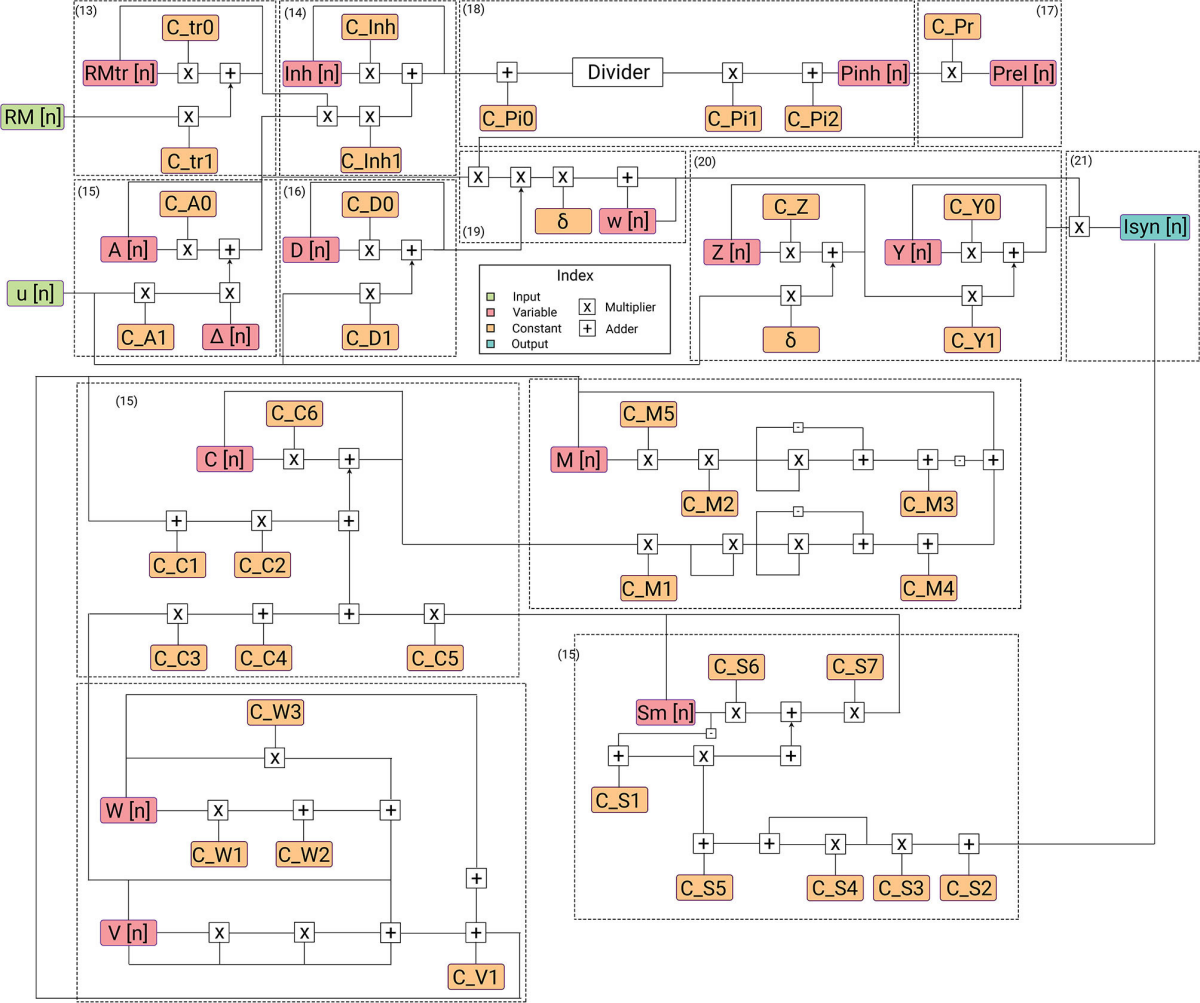
Description of flow of data in hardware architecture. Each submodule for its corresponding equation is highlighted. The architecture has the lowest power requirements for the complexity of the designed model.

**Figure 5:**
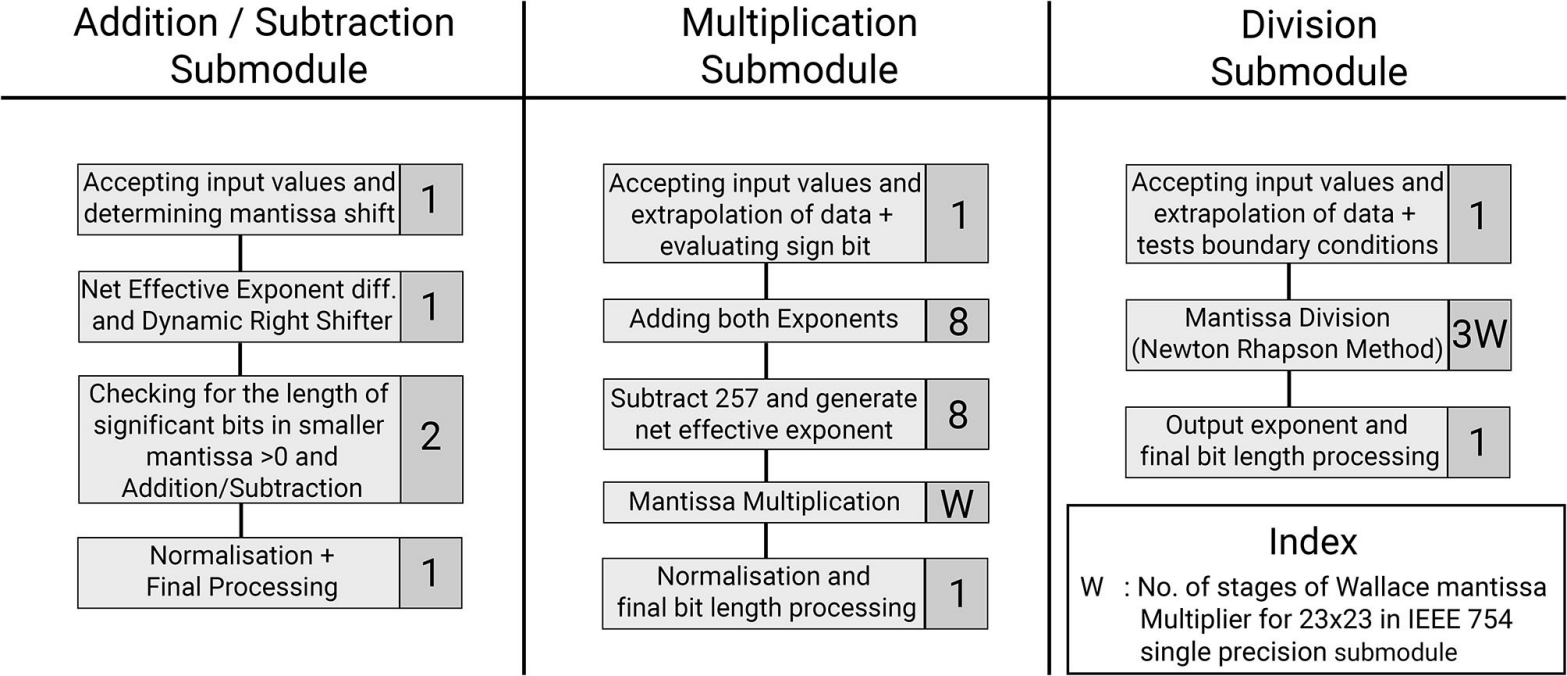
Optimization of the hardware module pipelining stages to obtain 1 GHz frequency. Represented are the three arithmetic submodules used in the SyNC architecture. Each task is represented with the number of pipelined stages involved for that task.

This section provides a detailed description of the hardware realization of the proposed discretized SyNC model. The computation core has been developed to work with IEEE-754 single precision floating point number system. The pathways of information flow in the hardware realization of SyNC are described in Figure 4. Figure 5 describes the different stages of pipelining in terms of registers or flip-flops in each arithmetic unit used in the model. The design has been pipelined extensively and even arithmetic operators have been pipelined for mantissa operations by using custom-made, non-compressed, tree structured, pipelined, moderately large sized and power consuming high speed, wallace tree multipliers. The largest combinational unit floating point multiplier architecture is a mantissa multiplication of respective mantissa widths, whereas the largest one in division is the iterative Newton-Raphson division method which consists of three mantissa width multiplications for both posit and float. All of these multiplications are done using custom implemented wallace tree unsigned multipliers for gaining greater speed and to meet acceptable operating frequency.

Design has been pipelined with basic pipeline registers and clock speed has been achieved as high as 1 GhZ in 45 nm technology node. The hardware design of the SyNC model, with exact same input configurations, are simulated in Questasim 10.0b Simulator by simulation scripts and output results are functionally verified by self-developed utility scripts in C++ v.11 and Python 3.8. For simulation as well as the hardware design for the given system of equations, we consider a constant input of retrograde messenger from the pre-synaptic region such that it is always just above the threshold for activation of synaptic plasticity parameter. We have implemented SyNC in RTL which are then mapped and synthesized using Synopsys Design Compiler on 45 nm OpenNangate technology. The power consumption is derived using Synopsys Power Compiler. Standard operating frequencies of almost 1 GHz is met for all the designs in 45 nm ASIC.

For low power, economical and optimized implementation of most widely used single precision float, we have made use of reduced complexity hardware of base arithmetic units. Complexity of other numerous subunits are also significantly reduced. This brought down the power and area footprints by orders of magnitude than the base version to 2.135 W and 1.877 *mm*^2^, respectively, by reduction of the number of intermediate registers and less frequent switching of intermediate variables. Through extensive pipelining, single precision floating point optimized version is able to meet acceptable operating frequency of 1,030 MHz. The operation parameters are described in Table 2.

**Table 2:** Operation parameters of SyNC architecture.

**Sl.No**.	**Parameter**	**Value**	**Units**
1.	Area	1.877	*mm*^2^
2.	Dynamic power	2.135	Watt
3.	Cell leakage power	32.1054	mW
4.	Total cell power	2.167	Watt
5.	Total no. of gates	2.35	Million
6.	Total no. of transistors	9.4	Million
7.	Speed-up by pipelining	3.88	N/A

One of the key features of our mathematical design is that an endocannabinoid feedback mechanism is involved. While this is a staple in analog designs, for hardware implementation, an inadvertent delay is obtained in the system. This is observed to be equivalent to 25 clock cycles in the hardware domain. Here, instead of resorting to Verilog AMS, we control the accuracy to the model and minimize this delay to negligible extent by varying δ. The role of δ in our model is defining the relationship between the clock timing and the actual timeframe of operation. Hence by increasing δ, we decrease the effective influence of the delay in the timeframe of operation of the synapse.

#### 4.1.1. Considerations for Error vs. Complexity in Architectural Design

The primary focus for development of a hardware model is for future use in large scale hardware implementations. Such applications necessitate low power consumption designs. Solutions such as linearization have been applied to obtain the required level of biological complexity but with reduced mathematical complexity. In the variable outputs obtained from the hardware realization of the SyNC neuron model, a latency in the results is observed. The net latency across all variables is 0.031 s, which is an effect of pipelining as well as the delay in feedback of retrograde messenger to the pre-synaptic region. As can be observed from the equations, while other equations have some garbage value output before the system stabilized for accepting action potential input, the only variable that does not always return to rest and which impacts the variables that come after it is synaptic weight, ω. For this very reason, the first values obtained just during the initialization of the model within this period must be rejected and the value of ω is fixed at 0, to remove the effect of default values of ω from influencing the nature of the curve. The error is <1% for architectures designed, which are sufficiently low and the output is barely affected and hence acceptable.

#### 4.1.2. Device Sensitivity Compared to Mathematical Model

For our hardware design with precision mode IEEE-754 Single Precision floating point, we observe that the amplitudes of the variables are higher in the hardware realization of our models than that of our software results. This is a result of using differential forms of the equations to develop the SyNC hardware realization. The response of the variables in the current curves is a consequence of the choice of value of δ for model implementation. The smoothness and root mean square (RMS) accuracy of all obtained curves are directly proportional to δ. Hence, we designed our hardware model with such considerations in mind. We can't increase the value of δ in our model because such an adjustment will reduce the speed-up achieved by the system.

However, for a biological system, the currency of communication is not entirely the form of the curve, but also the concentration of calcium influx associated with it. Rather, after RMS error reaches a particular range, it is this concentration influx which is more important to replicate. This can be evaluated by obtaining the error in area under the curve in the form of Area Average Error (AAE). Since for the SyNC neuron model, the conservation of molecules involved is of higher importance from a biological perspective, we disregard the amplitude error in favor of lower AAE after RMS error is within 1%.

### 4.2. Software Analysis of Autism Spectral Disorders

#### 4.2.1. Designing Mathematical Modules for ASD Scenarios in Extended Model

##### 4.2.1.1. FXS Syndrome

For modeling the effect of FMRP in FXS syndrome in a synaptic device, we consider its impact on mGluR5 LTD. We define the equilibrium dynamics for the mGluR5 due to accelerated generation of Homer 1a. The synaptic current influx in the postsynaptic region can be considered a directly proportional to mGluR5 activity. Since the increased sensitivity in dynamics is observed, the gain achieved can be expressed as:

(26) $$\begin{array}{l} \frac{dInflux}{dt}=-k_{fmrp}.k_{t}.\left[Isyn\right]\\ \;Isyn^{\prime }=Isyn+Influx \end{array}$$

Here, the dynamic parameter *k*_*fmrp*_ is the outcome of molecular stochastic process. Therefore, it behaves as the source of noisy behavior in this model for FMRP concentration. The variations in FXS are expressed first in secondary mediator in our model.

##### 4.2.1.2. Tuberous Sclerosis Complex

**Figure 6:**
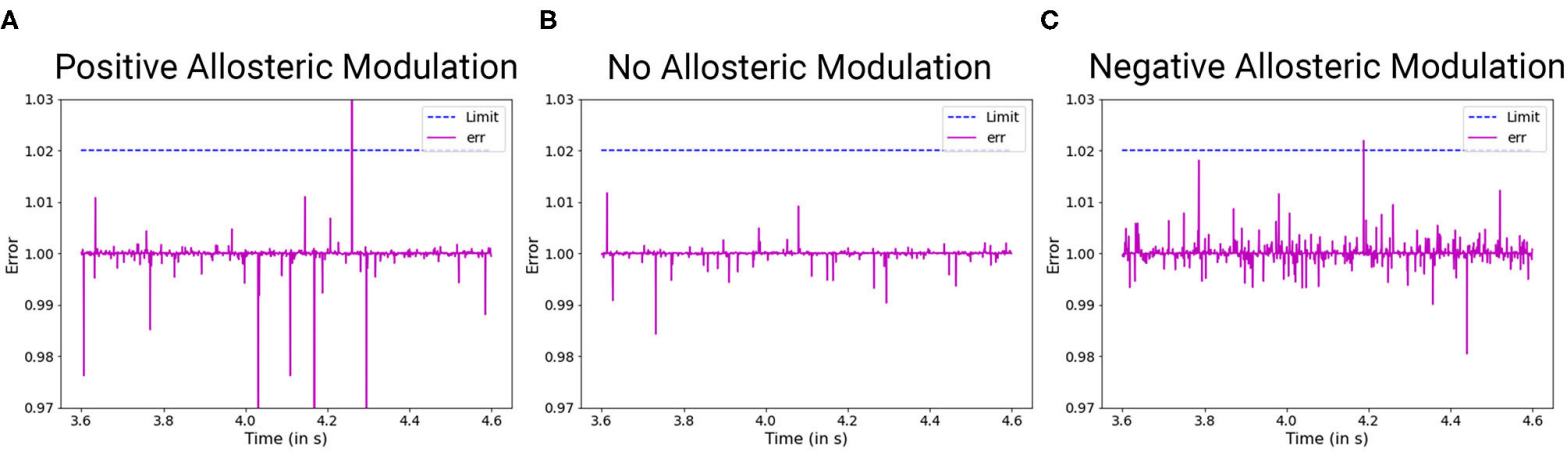
The effect of allosteric modulation in TSC. The limitations for positive allosteric modulation **(A)** and negative allosteric modulation **(C)** are compared with an unperturbed scenario **(B)** in which no allosteric modulation agents were introduced into the system. **(A)** Positive allosteric modulation results in higher amplitude and low frequency filter. **(C)** Negative allosteric modulation results in lower amplitude threshold and high frequency filter.

To understand and model TSC, we approach it from the perspective of the solutions that have been suggested in the literature. Allosteric modulation has been one of the most promising solutions that have been studied extensively in the recent times. Due to lack of an exact model of how both enhancement and inhibition of mGluR5 activity improves synaptic output in allosteric modulation of TSC (Figure 6), we hypothesize a mathematical form for the same that can duplicate such behavior successfully. Hence in our model, we consider the behavior of the NAM akin to lowering the threshold of mGluR5 activation, leading to easier activation of the post-synaptic region and improving mGluR-LTP. We consider the equivalent mechanism of the action of PAM by considering the impact of impaired protein function behavior onto the output dynamics of the synapse.

As observed from numerous studies, the activity of TSC is essentially centered around the activity of mGluR5. This is consistent with mGluR5 PAM alleviation of LTP by compensating for reduced mGluR5. However, mGluR5 must also be the primary carrier of distortion in this system because reduction of mGluR5 activity by NAM is also beneficial for the plasticity of the system. Hence, we hypothesize that the impaired protein synthesis is the generator of distortion in the process. We develop the equilibrium constructs of the system as follows:

(27) $$\begin{array}{l} \frac{dk_{mGluR5}}{dt}=+k_{mGluR5}-k_{namGluR5}\\ \frac{dk_{mGluR5}}{dt}=+k_{mGluR5}+k_{pamGluR5}\\ k_{namGluR5}>k_{mGluR5}>k_{pamGluR5} \end{array}$$

##### 4.2.1.3. NMDAR and Partial Agonist

D-cycloserine's activity as a partial agonist can be best described as a competitive process at NMDAR. Electrodynamically, in the concerned scenarios, the concentration of D-cycloserine is more than the concentration of NMDAR, hence the molecules unable to bind with NR1/NR2C receptors act as agonists. However, it is also observed that this effects the glutamate binding and hence does not affect the plasticity from its own path. The resultant glutamate binding is inhibited at NR2A, NR2B once all sites of NR2C are occupied by either of the agonist. It must be noted that D-cycloserine in natural state is not an action potential activated pathway involved in synaptic transmission dynamics, hence making it the noise source in the system. To demonstrate the role of D-cycloserine, we model its role in synaptic transmission dynamics via the competitive concentration dynamics process. The effect of such dynamics can be first observed in our model at the secondary mediator, the first state variable evaluated at the post-synaptic region and is directly dependent to mGluR5 activity. To better understand the process dynamics, the distortion for this specific case is evaluated by comparison between the simulated outputs to a noisy signal for the case where D-cycloserine is acting passively along with primary neurotransmitter (glutamate).

For the scenario involving D-cycloserine along with glutamate:

(28) $$\begin{array}{l} \frac{dIsyn}{dt}=\left[+\frac{Isyn}{k_{Glutamate}}-\frac{N}{k_{D-cycloserine}}+\frac{1}{k_{adjust}}\right] \end{array}$$

##### 4.2.1.4. Shank and Neuroglin

The role of Shank proteins in synaptogenesis and function (Sala et al., 2015) while understood to a certain extent, have not been successfully generalized due to the numerous ways Shank proteins can impact the synaptic function. Many experimental studies have been made to understand its role, but the highly diverse and often contentious conclusions from these studies have been an impediment. Here, we seek to reduce the core properties of the Shank such that all such scenarios can be simulated successfully. The core properties of Shank protein can be reduced to variations in NMDAR activity, NMDAR mediated LTP and NMDAR/AMPAR ratio at the postsynaptic region. We can model this condition by varying the equilibrium constant value of k or kNMDAR. To be able to model a wide range of NMDAR/AMPAR values, we adopt a working assumption that synaptic vesicle release is unbounded. We also consider the action of Neuroglins, synaptic cell adhesion molecules, which have effects complementary to that of Shank, as in the ratio of AMPAR/NMDAR becomes lower. We can hence develop a composite model that can effectively demonstrate the impact of Shank and Neuroglin dynamics on the effective change in synaptic current and the impacted changes can be observed in the secondary mediator dynamics.

(29) $$\begin{array}{l} \frac{Isyn^{\prime }}{Isyn}=\frac{k_{Shank}}{.k_{Neuroglin}}.k_{adjust} \end{array}$$

##### 4.2.1.5. Chromosome 15 Syndrome

A “chromosome 15 phenotype” is characterized by ataxia, language delay, epilepsy, intellectual disability, repetitive movement disorders, and facial dysmorphic features and has been described in individuals with chromosome 15 duplications (Muhle et al., 2004). We model the chromosome 15 inhibitory receptor action on neuronal dynamics onto the synaptic mediator and observe the effects on calcium dynamics in the post-synaptic region of the neuron.

#### 4.2.2. Classification of Noise Response to Steady Synaptic Output Potential for ASD Scenarios

**Figure 7:**
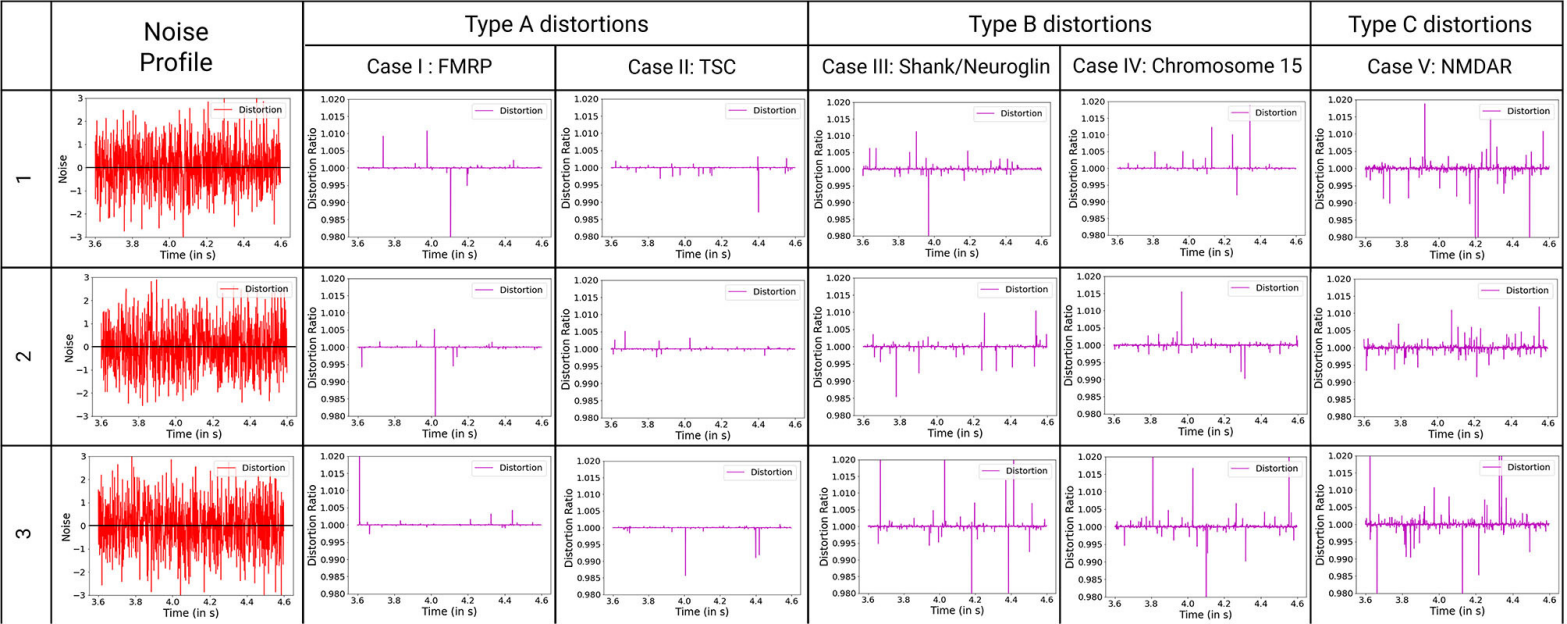
Classification of ASD mechanisms considered based on distortion from the ideal synaptic output potential, with ideal distortion response set at 1. Noise describes the variations in variables that are considered for these synaptopathies, with baseline for no noise input set at zero. The baseline for noise is added to the plots for reference. D-cycloserine here acts as partial agonist on NMDAR. Type A synaptopathies are categorized as the least amount of distortions. Multiple neurons must be affected by these particular synaptopathies to be observable. Type B synaptopathies have observable impact on the neuronal circuit even when occurring in a small collective of such affected neurons. Type C synaptopathies show abruptly high distortions that can lead to easily observable variations in the neuronal circuit.

Here, we observe the normalized impact of an identical noisy input to the synaptic potential output at the post-synaptic region of the neuron. The effective distortion observed in the synaptic output potential can be classified into three primary types (Figure 7). Type A shows the least amount of distortions. The impact on one synapse can be considered unobservable by experimental methods. Hence, we can say that to have observable impact on the neuronal circuit, multiple synapses and neural networks with them must be affected by these particular synaptopathies. Type B events show considerable distortions whose impact can be considered observable via high precision experimentations. To have observable impact on the neuronal circuit, a small collective of such affected synapses and networks are sufficient. Type C shows abruptly high distortions whose impact on a single synapse can be considered observable by experimental methods. Few such altered synapses for neurons in key processing regions of the brain will lead to observable variations in the neuronal circuit.

## 5. Discussion

In this work, we realize a biologically descriptive synaptic model for neural communication, with primary focus on modeling the complexity of a synapse in a way that can translate into competent hardware models. Large scale implementation of such models is aimed at utility in designing biologically extensive neuronal circuits. We focus on Autism Spectrum Disorders in which a variety of synaptopathy mechanisms operate in various syndromes. By generating a set of ASD interrogation algorithms (ASDint) we modeled changes to synaptic dynamics in regard to corresponding disease variables and observed the spread of their impact in larger neural circuits. This is the first demonstration of algorithms designed specifically to address complex synaptopathies of ASD. Co-development of ASDint over the SyNC core algorithm provides a powerful new computational and hardware approach to benefit ASD experimental analysis and to help predict manifestation of symptoms for ASD. This is accomplished by abstraction of neuronal behavior at the synapse while describing the complexity involved in the interactions of the implicated primary state variables. Importantly, such an approach is not restricted to ASD and the SYNC algorithmic core can be used to design network models for any such synaptopathies with complex dynamics.

Most of the synapses in the CNS are low pass filters. While the synaptic communication properties are not ideal, the attenuation is not significant. For a large network of neurons consisting of many synaptic nodes, it is important that the signal amplitude is not attenuated to a large degree or else distant synaptic nodes in the neural communication framework won't be able to communicate with each other. Synapses modulate not only the amplitude of the action potential but also the selectivity and accuracy of the synaptic output response. The filter form of synapses has decisive implications on their noise response (Voglis and Tavernarakis, 2006; Faghihi and Moustafa, 2015). Synapses as such can be widely classified into three such classes: high pass, low pass and band pass filters. Those synapses with release probability below 0.3 act as high pass filters (Goda and Südhof, 1997). Such synapses have very high selectivity and noise resistance to environmental procedures. However, the attenuation to synaptic output potential is also very significant in such synapses. Synapses with initial probability of neurotransmitter release greater than 0.7 act as low pass filters (Murphy et al., 2004). Such synapses have very low selectivity, while attenuation to synaptic output potential is also rather insignificant in such synapses. However, the resulting synaptic transmission is affected by noise generated by environmental procedures to an observable degree. Synapses with intermediate initial release probability, between 0.3 and 0.7, act as band pass filters (Rose et al., 2013). In our models, we consider the initial neurotransmitter release probability of 0.5, which adequately describes a low pass filter glutamatergic synapse.

Synaptopathies can be described in the form of distortions observed in the system compared to normal conditions. The distortion obtained from the ASDint simulations show consistency with the conditions underlying those specific synaptopathies. Two primary ways to look at a noise response is the gain in potential strength they provide and the number of spikes in the resultant output that are at a threshold to impact the system dynamics. Gain in potential results in stronger and more sensitive LTP dynamics, reducing the threshold of action potential spike input and frequency required to activate it. Such an output response implies improved LTP dynamics and lower LTD threshold, resulting in greater sensitivity of the model as well as lower duration of information retention of the synapse. Greater noise spikes result in false activation of the region and can result in faulty activation of the boutons. Such impulses can result in variations in synaptic weight and lead to repetitive behaviors. In analysis of the ASD mechanisms different resulting distortions occurred.

Evaluation of distortions provides insights into phenotypes observed in ASD synaptopathies. For TSC under the impact of negative allosteric modulation, the noise spikes in the distorted curve, although of lower amplitude threshold, are greater than the general scenario. This can manifest itself in the form of false activation of the post-synaptic region, which can be considered a trigger for repetitive behaviors. When positive allosteric modulation occurs, we observe the distortion curve to have higher noisy spikes as well as lower attenuation of action potential. Hence, it has better LTP characteristics and LTD threshold that can be associated with obstructions in learning and lower attention span. Modeling the FXS syndrome in ASDint module, we observe that LTP is very easily activated here and can be observed in the form of lower LTP threshold. Thus, it can be associated with difficulty in learning process as well as increased sensitivity to noisy inputs in the neural circuitry. The interactions of partial agonist with NMDAR show us the output of competition in the synaptic channel manifested at the synaptic gate. Social withdrawal and repetitive behavior are associated with this particular synaptopathy. On observing the noise response of the ASDint module, we see that the noise-induced variations are the highest when compared to the other three scenarios and can be considered a trigger for repetitive behavior. For Shank, NG activity variations, the NMDAR induced LTP is affected and results in higher required amplitude of LTD, which we can see from the noise plots resembles the expected activity variations. Chromosome 15 syndrome is associated with repetitive movements which as observed from the distortion curve, could be a consequence of false activation of post-synaptic regions.

Large scale hardware usage is key for further understanding the impact of plasticity and synaptopathy mechanisms on larger circuits. In our hardware modeling, use of the IEEE 754 single precision floating point has accuracy within tolerable error ranges. It is also remarkably small and efficient in terms of PPA (power, performance, area) vs. ARM core implementations. It has been a prevalent practice of late to use floating point numerals in 16 bit widths or lower order custom width floats to optimize computational resources specifically for edge computing use-cases. Such practices give us higher power efficiency which increases the scalability of the neuronal circuit model. We were unable to consider them without decisive loss in accuracy within the neuron ASDint model. Due to such considerations, IEEE 754 single precision floating point is noticeably the most suitable bit width for our application. For comparison of our neural network, one of the well known state-of-the-art SNN architectures, IBM TrueNorth (Akopyan et al., 2015) and Intel Loihi (Davies et al., 2018) has been considered. IBM TrueNorth uses a smaller technology node of 28 nm and consumes about 100 mW of power. It has a power density of 20 mW/*cm*^2^ consisting of 5.4 billion transistors. Intel Loihi has used a further smaller node of 14 nm processor with a 2 billion transistor size or 60 *mm*^2^ in chip area. The proposed SyNC architecture is designed on a technology node of 45 nm and consists of 9.4 million transistors over an area of 1.877 *mm*^2^. Hence, we can observe that we are able to bypass the requirements of programmability to model diverse descriptive synaptic models by design of a minimalist ASIC synaptic core that describes every state variable in a synapse. On utilization of lower technology nodes, we expect performance of our device to be at par with state of the art SNN architectures. For future studies a programmable Application Specific Processor like the former chips are being targeted for better flexibility and programmability and to have a one chip for all solutions.

Models built in equivalent Posit numeral representation, such as Jaiswal and So (2019) and Chatterjee et al. (2021) have demonstrated highly flexible and fast convergence in specific format scientific functions. Such models have shown accuracy comparable to IEEE 754 double precision floating point, using much lesser resources than the latter. However, due to complications in evaluations that result from dynamic power owing to higher switching of states, the net power requirement is significantly high on account of deep pipeline and conversion of FPGA fabric optimized design to general ASIC.

## Conclusion

The SyNC model provides us with an elaborate and accurate neuron model. It is one of the only hardware models that has efficiently used posit in highly sensitive systems, while achieving low error percentages. Its low power and low error margins make it optimal for large scale digital implementations. Finally, ASDint and SyNC can be used to process and test data obtained from experiments and hypothesize neuron network possibilities and hence identify key variables and areas for experimental verification. Hence, when used in large scale network models, SyNC accelerates our understanding of the roles of synapses, synaptic plasticity and neuronal circuits in brain function.

## Data Availability Statement

The datasets presented in this study can be found in online repositories. The name of the repository and accession number are found at this link: https://github.com/RnkC/SyNC.

## Author Contributions

AM and JP conceived of the project and experimental design and helped to generate algorithms and analyzed the data, and wrote the manuscript. AR supervised all aspects of the hardware design and realization. RC generated the software algorithms, performed the ASD analysis, and developed ASDint algorithms for case study. SC and SM developed and deep pipelined the SyNC model in relation to the algorithms with assistance from RC. RC wrote the initial manuscript and generated figures and tables. All authors contributed to the article and approved the submitted version.

## Conflict of Interest

SM is employed by the company Samsung Electronic. AR is employed by the company Intel Corporation. All authors have a patent pending for software/hardware related to this study.
